# Bedtime App–Guided Mindfulness Meditation in Patients With Insomnia: Mixed Methods Feasibility and Acceptability Pilot Study

**DOI:** 10.2196/67366

**Published:** 2025-09-30

**Authors:** Yan Ma, Peter M Wayne, Janet M Mullington, Suzanne Bertisch, Gloria Y Yeh

**Affiliations:** 1 Osher Center for Integrative Health, Division of Preventive Medicine, Brigham and Women’s Hospital, Harvard Medical School, 75 Francis St, Boston, MA, 02215, United States, 1 (617) 732-8544; 2 Department of Neurology, Beth Israel Deaconess Medical Center, Harvard Medical School, Boston, MA, United States; 3 Division of Sleep and Circadian Disorders, Department of Medicine, Brigham and Women's Hospital, Harvard Medical School, Boston, MA, United States; 4 Division of General Medicine, Beth Israel Deaconess Medical Center, Harvard Medical School, Boston, MA, United States

**Keywords:** sleep, insomnia, mindfulness, meditation, smartphone, app, feasibility, remote delivery, bedtime, mixed methods

## Abstract

**Background:**

While mindfulness meditation (MM) apps have gained popularity as a tool for promoting sleep, research focusing on bedtime mindfulness practice and app usage is limited.

**Objective:**

As the first step toward understanding the efficacy and mechanisms of such bedtime practice and to inform future investigations, the goal of this pilot study was to explore the feasibility of app-guided bedtime MM practice with both in-lab and at-home physiological and self-report sleep remote assessments.

**Methods:**

We conducted a single-arm, prospective mixed methods pilot study that included both standard in-lab sleep studies and remote at-home assessments of individuals with insomnia disorder with self-reported difficulty falling asleep. Participants practiced MM guided by a commercially available smartphone app at bedtime for 4 weeks. Pre-post assessments included a battery of sleep-related and psychological health questionnaires, objective physiological sleep measures (polysomnography and actigraphy), and daily sleep logs. We also conducted qualitative exit interviews to further assess feasibility and acceptability. Transcripts were analyzed for dominant themes using inductive and deductive qualitative methods.

**Results:**

We recruited 13 participants with chronic insomnia (symptoms ≥3 nights weekly for ≥3 months) to complete the study protocol within 8 months (retention rate 77%). We were able to collect analyzable physiological and psychometric data with overall completion rates of more than 90%. The study was deemed feasible, meeting a priori benchmarks including recruitment, retention, completion, and adherence. The 10 participants retained in the program had excellent engagement (95% completion of in-lab studies, 100% completion of questionnaires, and 91% compliance with use of the app). Our preliminary analysis of subjective measures indicated improvement in sleep quality, insomnia severity, and presleep arousal, including Pittsburgh Sleep Quality Index change of −3.7 (95% CI −6.7 to −0.7), Insomnia Severity Index change of −4.5 (95% CI −7.7 to −1.4), Pre-Sleep Arousal Scale change of −7.7 (95% CI −13.1 to −2.3), and trend toward improvement in the Ford Insomnia Response to Stress Test indicated by a change of −2.5 (95% CI −5.9 to 0.9). From qualitative data, we identified domains that inform the feasibility and acceptability of the study, including (1) barriers to sleep prior to the study, (2) benefits and skills imparted by mindfulness, and (3) feedback on app use. Benefits and skills imparted by mindfulness included decreased catastrophizing, acceptance and nonreactivity, body awareness and relaxation, self-kindness, awareness of sleep hygiene and bedtime routine, earlier defusing of stress, increased focus and presence, and calm throughout the day.

**Conclusions:**

Bedtime app-guided MM as an intervention in patients with insomnia and the hybrid study design with in-lab and at-home assessments are feasible and acceptable. This study informs the design of future clinical and mechanistic research examining app-guided MM to impact insomnia severity and presleep arousal.

## Introduction

Insomnia affects up to 30% of the population with a total public health cost of US $107 billion annually in the United States [1-4]. Insomnia disorder accounts for more than 5.5 million visits to physicians each year [5] and is associated with a wide range of deleterious health consequences including cognitive difficulties, poor work performance, and decreased quality of life, as well as increased risk of hypertension, diabetes, obesity, anxiety, depression, heart disease, stroke, and all-cause mortality [6,7]. Sleep medications can produce adverse effects and have risks, including fall risks, dependence, and potential abuse, which vary by the classes of medicine [8-11]. Clinical guidelines [12-14], in fact, recommend nonpharmacological therapy (cognitive behavioral therapy for insomnia) as first-line therapy (Grade 2B); however, this is underutilized and often not available. Even with emerging cognitive behavioral therapy for insomnia apps, limited availability, overgeneralized advice, usability, limited scope of interventions, insufficient clinical evidence, patients’ engagement, and self-discipline are common issues. Results from the National Health Interview Survey showed that 49% of responders reported sleep troubles (n=26,742 adults), and mindfulness meditation was one of the most commonly used mind-body approaches [15]. Recent studies support mindfulness-based interventions as a promising nonpharmacological intervention for reliable and durable improvement of sleep quality, including among patients with insomnia disorder [16-20]. However, the existing evidence on sleep improvement is limited due to heterogeneity, lack of rigor across studies, and poor understanding of underlying mechanisms [19,21,22].

Models of insomnia often include cognitive arousal, hyperarousal, rumination, and worry. In clinical settings, however, many patients with insomnia commonly complain of inability to fall asleep due to “racing thoughts” [23-25]. This more colloquial term has been described as a subjective acceleration and overproduction of thoughts, which is highly associated with affective dysregulation (ie, cyclothymic temperament traits) and hyperarousal [26,27]. “Racing thoughts” has been shown to be increased in the evening and at bedtime in patients with sleep-onset insomnia. Importantly, “racing thoughts” at bedtime, but not rumination and worry, was associated with insomnia severity [28]. Under this high mental load, the sense of urgency about falling asleep adversely affects sleep onset latency [29]. Well-established evidence has shown that people with presleep hyperarousal are prone to difficulty initiating and maintaining sleep at night. Mindfulness meditation (MM) may be a promising nonpharmacological treatment for improving sleep quality by reducing bedtime stress and arousal [30], while barriers to adoption and scalability are also issues.

Technology-based approaches facilitate remote delivery of mindfulness-based interventions to be more affordable, accessible, and scalable. The increased popularity of mobile apps has the potential to increase access, assist home practice, and enhance care after formal in-person treatment has concluded. In practice, there has been a surge in the use of commercially available mindfulness-based smartphone apps as alternatives to formal interventions. In the case of mindfulness and insomnia, a survey from 12,151 paying subscribers of a commercially available mindfulness app Calm showed that 90% of subscribers have sleep difficulties, 77% of users download the app primarily for improving sleep, and the most common reasons for sleep disturbances were racing thoughts (7084/8604, 82%) and stress or anxiety (6307/8604, 73%) [31-33].

While MM apps have gained popularity as a tool for promoting sleep, there are no established guidelines on how best or when to use these tools. While the number of people who tend to use apps at bedtime is rapidly increasing [31,32], specific research focusing on bedtime app usage is limited, and the efficacy and mechanisms of such bedtime practice are unknown. In fact, bedtime screen use is often discouraged with typical sleep hygiene strategies. Many apps now target sleep difficulties and have begun to incorporate sleep-specific content but the research is deficient. Among the small number of clinical trials using remote-delivered mindfulness for sleep, there are several gaps. For example, most study the use of these programs during the daytime with no studies explicitly examining bedtime practice. Within this context, we tested the feasibility of a clinical trial involving an app-based bedtime MM intervention and both in-lab and at-home assessments. This pilot study lays the foundation for future studies to better understand bedtime app-guided MM and explore temporal correlations of bedtime practice and subsequent sleep quality.

## Methods

### Study Design

We used a single-arm prospective study design investigating a 4-week app-based bedtime mindfulness intervention in individuals with chronic insomnia disorder with self-reported difficulty falling asleep that included pre-post measurements, both standard in-lab and remote at-home assessments. We have followed reporting guidelines from the CONSORT (Consolidated Standards of Reporting Trials) pilot extension except for those criteria specific to randomized controlled trials (RCTs).

### Ethical Considerations

This study was registered at ClinicalTrials.gov (NCT04242771). The Institutional Review Board at Beth Israel Deaconess Medical Center approved this study. Participants were provided with a copy of the consent form by email before the consent discussion. Initial screening and informed consent procedures were done remotely. Signatures were obtained using REDCap (Research Electronic Data Capture) e-signature, prior to the conduct of any research procedures. To guard against concerns of signature legitimacy, the signature will be obtained live during the consent appointment. A PDF of the electronically signed consent form was provided to the participant by email and was retained in the study research file. Only the primary investigator and a key co-investigator have access to participant information. All identifiers were transferred to a password-protected database. Our study database did not include data items that uniquely identify participants such as subject name, address, phone number, social security number and medical record number, but will be indexed by a study ID number. After all interviews were complete, the recordings were transcribed verbatim and stored in a password-protected file. The transcriptions only had coded information without any participant identifiers. Participants were compensated up to US $200 for study participation (US $50 after the first visit and US $150 after completion of the study). Participants were also reimbursed for 1-year Calm app charges and parking expenses for each visit.

### Eligibility Criteria

We included adult participants who met the following inclusion criteria: (1) age 20‐50 years; (2) chronic insomnia (based on common key criteria from *Diagnostic and Statistical Manual of Mental Disorders* [Fifth Edition] [*DSM-V*] and International Classification of Sleep Disorders [ICSD-3]) with any complaints of difficulty initiating sleep, difficulty maintaining sleep, early morning awakening, and the frequency (≥3 nights per week) and duration (≥3 months) despite adequate sleep opportunity and an appropriate sleep environment, they had self-reported sleep onset latency >20 minutes; (3) speak and understand English; and (4) have a smart device for mobile app installation. Exclusion criteria included (1) those with sleep disorders other than insomnia (eg, narcolepsy, restless leg syndrome, insufficient sleep syndrome, jet lag, etc), (2) shift workers or having routine night shifts, (3) women who are pregnant or breastfeeding, (4) those with a history of head trauma or surgery, (5) those with regular (defined as twice a week or more) practice of mind-body interventions, (6) those with neurological disorders (eg, epilepsy, dementia, stroke, Parkinson disease, neuroinfections, brain tumors, etc), and (7) those with current use of neurologic, psychiatric, or cardiovascular medications that have expected neuropsychiatric or cardiovascular effects (ie, β-blockers, hypnotics, and antidepressants).

### Recruitment and Consent

We recruited participants through fliers posted at local libraries and community centers in and around Boston and through ResearchMatch.org, a public-facing institutional review board–approved web-based recruitment tool. Interested individuals contacted us via email or phone. Eligibility was then assessed over a phone screen. Screening interviews were conducted by a research fellow trained in sleep medicine with both clinical and research experience working with patients with insomnia. Potential participants were explicitly informed that participation in the research study is voluntary. We obtained full informed consent during a consent appointment via phone, and signatures were obtained using REDCap e-signature in real time. A PDF of the electronically signed consent form was provided to the participant via email with REDCap secure link and code.

### Interventions

Participants were asked to download a commonly used and commercially available mobile app (Calm), which was available for both Android and iOS systems. We instructed participants to practice MM using the specific sleep programming available within the app every night at bedtime for 4 weeks (weeks 1‐4). In the first week of intervention (week 1), participants followed an introductory program, “Seven Days of Sleep,” which included 7 soundtracks (12‐15 minutes each) for guided MM practice. This practice involves techniques such as breathing exercises, mental imagery, awareness of body and mind, and muscle and body relaxation. During weeks 2‐4, participants repeated the same sessions or chose their preferred programs under “Meditation” within the “Sleep” section. Participants could choose from a list of 28 programs with a total of more than a hundred audio tracks of various time lengths (from about 5 minutes to more than 1 hour). Participants were instructed to practice a minimum of 10 minutes but no more than 30 minutes at bedtime. We also provided a series of instructions to minimize the negative impacts from light exposure and the use of digital devices (eg, dim light on the screen, auto-stop playing after a set time duration, no-disturb mode, audio only without screen exposure, etc). There were no limitations for daytime practice or for use of other meditations available on the app.

### Questionnaires

Self-report measures were collected during participants’ baseline and postintervention visits. The battery of sleep-related measures included the following.

Pittsburgh Sleep Quality Index (PSQI) is a subjective sleep assessment, which includes multiple sleep-related variables over the preceding month. The PSQI yields 7 component scores: subjective sleep quality, sleep latency, sleep duration, habitual sleep efficiency, sleep disturbances, sleep medication, and daytime dysfunction. Component scores range from 0 to 3 and are summed to obtain a global score, which ranges from 0 to 21. A score of >5 suggests poor sleep quality, and higher scores suggest greater sleep disturbance [34].

Insomnia Severity Index (ISI) is a brief instrument that assesses insomnia according to the criteria from the *Diagnostic and Statistical Manual of Mental Disorders* (Fourth Edition) (*DSM-IV*) and the International Classification of Sleep Disorders. Its reliability, validity, and sensitivity to treatment response have been documented in the general population and with patients with insomnia [35,36]. A 5-point Likert scale is used to rate each item (eg, 0=no problem; 4=very severe problem), yielding a total score ranging from 0 to 28. The total score is interpreted as follows: absence of insomnia (0‐7), subthreshold insomnia (8-14), moderate insomnia (15-21), and severe insomnia (22-28).

Ford Insomnia Response to Stress Test (FIRST) questionnaire is a 9-item self-report measure of trait vulnerability to sleep reactivity [37,38]. The FIRST has shown acceptable reliability and validity, is predictive of future sleep dysfunction, and has been related to objective measures of sleep reactivity. The total score ranges from 9 to 36. High scores on the FIRST indicate greater vulnerability to sleep disruption.

Pre-Sleep Arousal Scale (PSAS) is a widely used measure to evaluate presleep arousal [39]. It is a 16-item scale that measures 2 distinct domain subscores of cognitive and somatic arousal before falling asleep (range 8‐40, higher score indicates greater arousal).

Dysfunctional Beliefs and Attitudes about Sleep (DBAS) was developed to evaluate sleep-disruptive cognitions and purported maladaptive beliefs in insomnia. The validated brief version (DBAS-16) has a 10-point Likert scale ranging from 0 to 10. For each of the 16 beliefs, the number corresponding to the degree of belief (ie, 0=strongly disagree, 10=strongly agree) is circled. The DBAS-16 index score is a mean item score (ie, item scores are summed and divided by 16), and a higher score reflects greater dysfunctional beliefs about sleep [40]. DBAS-16 has been shown sensitive to several indices of changes with insomnia treatment and has been found reliable for discriminating between self-defined good and poor sleepers in both younger and older adults.

Generalized Anxiety Disorder 7-item Scale is a widely used diagnostic self-report scale for screening, diagnosis, and severity assessment of anxiety disorder. It measures anxiety symptom severity as a predictor of anxiety disorders (range 0‐21, higher score worse), 0‐4 indicating minimal anxiety, 5‐9 indicating mild anxiety, 10‐14 indicating moderate anxiety, and scores greater than 15 indicating severe anxiety [41,42].

Patient Health Questionnaire–9 (Depression Scale) is a validated measure of depressive symptoms. The 9 items are based directly on the 9 diagnostic criteria for major depressive disorder in the *DSM-IV*, with ratings from “0” (not at all) to “3” (nearly every day). The total score ranges from 0 to 27 and suggests the level of depression: 0‐4 minimal, 5‐9 mild, 10‐14 moderate, 15‐19 moderately severe, and 20‐27 severe depression [43,44].

Mindful Attention Awareness Scale (MAAS) is a commonly measured scale for measuring mindfulness in positive psychology. The MAAS measures an individual’s tendency to enter a state of mindfulness through the individual’s frequency of having certain experiences related to mindfulness and mindlessness. It includes 15 statements that respondents rate on how frequently they engage in the activities described, on a scale from 1=almost always to 6=almost never. Scores on these statements are then combined to create an overall score of mindlessness or mindfulness, with higher agreement indicating a lesser tendency to enter into a mindful state, while a lower score indicates a greater tendency toward mindfulness.

### Daily Logs

After the baseline visit, participants were asked to complete a sleep diary and MM practice log each morning to recall the previous night. The logs were completed daily during the 4 weeks of intervention. The sleep logs documented time to bed and waking up, total sleep time, number of overnight awakenings, overall score of sleep quality, and stimulant intake (eg, any coffee, alcohol, etc). MM practice adherence during the 4-week intervention period was tracked with a daily log format similar to the sleep logs, including frequency and duration of practice, strategies used (eg, breathing exercise, body scan, etc), and the names of the selected app programs or recordings.

### Qualitative Interview

All participants were interviewed postintervention using a semistructured interview to gather information about each participant’s experience with sleep prior to and during the study, as well as study and intervention feedback. Questions included both simple response (eg, Yes/No) questions focused on app acceptability and open-ended questions on feasibility or acceptability, barriers or preferences to practice, specific app feedback, and overall study experience. We further explored helpful components of the intervention and their relation to sleep. To ensure consistency, all the interviews were conducted by a single research team member (YM) who was trained in qualitative research and conducting interviews. Interviews were audio-recorded and transcribed verbatim. Two reviewers (YM and GY) independently reviewed the transcripts. Responses to Yes/No questions are reported as frequencies. For open-ended responses, we used a combined approach with both inductive and deductive methods to describe the data and extract emergent themes. With the deductive (theory-driven) approach, we used existing frameworks and hypotheses about how the intervention might help patients to seek support for specific themes within the data. With the inductive (data-driven) approach, we also allowed completely new patterns and themes to emerge from the data de novo.

### Objective Sleep Assessments

As part of our hybrid design, we conducted both in-lab and at-home remote objective sleep assessments. This included (1) standard, attended in-lab polysomnography (Natus SleepWorks; Natus Medical Incorporated) at baseline and then postintervention (week 5) at the Clinical Research Center at Beth Israel Deaconess Medical Center; (2) ambulatory heart rate monitoring 1 night per week on a typical night to include bedtime routine, MM practice, and overnight during sleep; and (3) actigraphy (Actiwatch 2s; Respironics Inc) for 2 consecutive weeks starting at the baseline visit, week 0 as baseline assessment, and week 1 as the first week of intervention. Participants were instructed to wear the watch continuously on the nondominant wrist. To minimize discrepancies between sleep log and actigraphy data, participants were asked to push the event marker just prior to falling asleep and upon first awaking every morning. All raw data of physiological signals were extracted. For these objective sleep assessments, this manuscript focuses only on feasibility and adherence metrics. Given the scope and depth of sleep physiological signal analyses, additional findings are planned in a separate manuscript that explores the mechanism of bedtime MM and association between physiological changes and subsequent night sleep quality.

### Feasibility and Acceptability Measures

This is a proof-of-concept pilot feasibility study. Primary analysis of data was limited to descriptive feasibility statistics. Assessment completion rate was defined as the number of valid data recordings divided by the total planned nights, that is, 5 nights of heart rate recording (1 in each week) and 14 nights of actigraph data. Compliance rate was calculated as the total number of nights of practice during the 4 weeks divided by 28. We hypothesized that (1) we would be able to recruit and enroll 12 patients with insomnia within 12 months, (2) we would retain >75% of participants in the program, (3) they would exhibit >70% compliance with use of the app, (4) participants would find the intervention acceptable based on qualitative interviews, and (5) we would be able to collect clean analyzable physiological and psychometric data from at least 80% of the participants.

### Statistical Analyses

Per guidelines for pilot trials set forth by National Center for Complementary and Integrative Health and others [45], we did not power this study for testing intervention efficacy or pre-post differences and thus emphasize descriptive statistics and data visuals here. The planned target of 12 participants was based on study needs and pragmatics (including time and budget considerations). Based on other pilot studies our group and others have conducted, this is a sufficient sample to evaluate feasibility metrics [46]. Statistical analyses were performed using IBM SPSS Statistics (version 25; IBM Corp). Continuous data are reported in text and tables with means and SDs for normally distributed data and with medians and IQRs for skewed data. Discrete data are reported as absolute numbers and percentages.  

## Results

### Feasibility Outcomes

**Figure 1.**
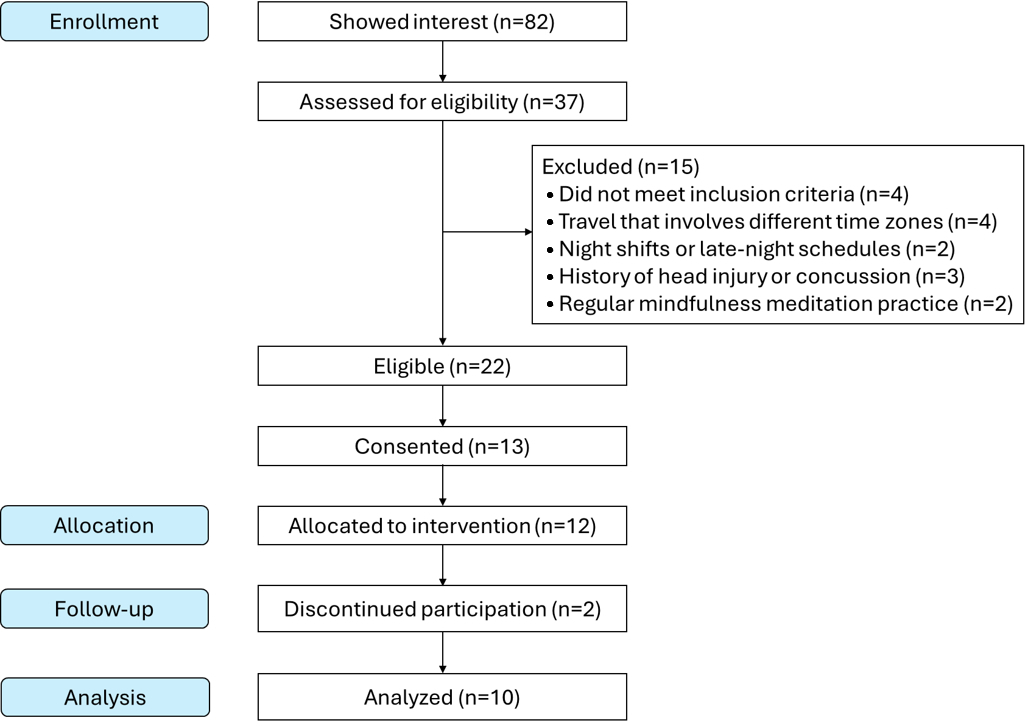
Modified CONSORT (Consolidated Standards of Reporting Trials) flowchart (single-arm nonrandomized study).

Recruitment started in December 2022, and data collection was completed in July 2023 (Figure 1). During the 8 months, we screened 37 interested individuals and were able to identify 13 eligible participants who consented to the study. The resulting consent-to-screening ratio was 13/37 (35%). Of these 13, 1 participant withdrew prior to any study procedures or testing (changed her mind regarding overnight sleep study). Two additional participants withdrew due to personal reasons (unemployment and schedule changes) prior to the start of intervention (with incomplete and complete baseline assessments, respectively). Ten participants completed all procedures including intervention and assessments (overall retention of 10/13, 77%).

### Demographic Characteristics

Full baseline demographic characteristics are shown in Table 1. The 12 participants for whom we have baseline data ranged in age from 22 to 50 years (mean age 30.42, SD 7.77years). There were no differences between those who enrolled (n=12) and those who completed the study (n=10) with respect to demographic characteristics (Table 1). The final sample of participants who completed the study were more women (7/10, 70%), relatively well educated, having insomnia for more than a year (7/10, 70%), full-time employed (7/10, 70%), and single (7/10, 70%).

**Table 1.** Baseline demographic characteristics of participants with chronic insomnia^a^.

Characteristics	All enrolled (n=12)	All retained and completed (n=10)
Age (years)	30.42 (7.77)	30.46 (8.15)
BMI	22.60 (2.35)	22.49 (2.45)
Sex, n (%)		
Female	7 (58.3)	7 (70.0)
Male	5 (41.7)	3 (30.0)
Race or ethnicity, n (%)		
White	5 (41.7)	4 (40.0)
Asian	6 (50.0)	5 (50.0)
Other	1 (8.3)	1 (10.0)
Education levels, n (%)		
Bachelor’s degree or below	7 (58.3)	6 (60.0)
Master’s degree or above	5 (41.7)	4 (40.0)
Insomnia duration, n (%)		
9 months to 1 year	3 (25.0)	3 (30.0)
<5 years	5 (41.7)	3 (30.0)
>5 years	4 (33.3)	4 (40.0)
Employment status, n (%)		
Full-time	7 (58.3)	7 (70.0)
Part-time	5 (41.7)	3 (30.0)
Marital status, n (%)		
Single	9 (75.0)	7 (70.0)
Married	3 (25.0)	3 (30.0)
Subjective sleep measures, mean (SD)		
Usual sleep-onset latency (minutes)	N/A^b^	78.75 (48.1)
Usual sleep duration (hours)	N/A	363.0 (43.5)
Objective sleep measures		
Sleep-onset latency (minutes), mean (SD)	N/A	44.0 (44.2)
Total sleep time (minutes), mean (SD)	N/A	357.8 (80.9)
Sleep efficiency, n (%)	N/A	80.5 (7.7)
Wake after sleep onset (minutes), mean (SD)	N/A	90.3 (48.7)

a Continuous data (age and BMI) are presented as mean and SD of the mean. Categorical data are presented as n (%).

b N/A: not applicable.

Participants reported an average of 4.3 nights per week with difficulty falling asleep and 4 mornings per week waking up early and still feeling tired or unrested. At baseline, on average, participants reported 101 minutes to fall asleep, 92 minutes staying awake at night, and 24.5 minutes to fall back to sleep after awakening at night. Most of the participants (7/10) had tried melatonin for sleep but did not find it helpful.

At baseline, all participants had poor sleep quality, indicated by PSQI scores ranging from 7 to 16. Insomnia severity, indicated by the ISI score, ranged from 8 to 22, with 6 (60%) participants having subthreshold insomnia, 3 (30%) participants having moderate insomnia, and 1 (10%) participant having severe insomnia. Based on the Generalized Anxiety Disorder 7-item Scale scores (ranged 3‐8 at baseline), participants had no (4/10, 40%) or mild anxiety (6/10, 60%). The Patient Health Questionnaire–9 scores (ranged 3‐8 at baseline) indicated that 3 (30%) participants had minimal depression and 7 (70%) participants had mild depression.

### Assessments Completion and Intervention Compliance Rates

All the 12 participants completed baseline in-lab sleep study. The 10 participants retained in the program exhibit an overall 95% completion of in-lab studies, 100% completion of questionnaires, and 91.4% compliance with use of the app (weekly compliance analysis shown in Table 2). On average, the completion rate of daily sleep logs was 100% at baseline week and 89.6% during the 4 weeks of intervention. Remote data collection by actigraph device and heart rate monitor showed overall 100% and 92% completion rates, respectively. At the end of the study, 9 of 10 participants completed their second in-lab study. One participant missed his postintervention in-lab sleep study due to extended travel out of the state. All of the 10 participants completed pre- and postquestionnaires and exit interviews.

**Table 2.** Assessment completion rates by study week.

Variables	Baseline (week 0)	Week 1	Week 2	Week 3	Week 4	Postintervention
In-lab sleep study	100%	N/A^a^	N/A	N/A	N/A	90%
Questionnaires	100%	N/A	N/A	N/A	N/A	100%
Bedtime practice	N/A	95.7%	90%	90%	90%	N/A
Sleep logs	100%	100%	100%	100%	100%	N/A
Heart rate monitoring	90%	90%	90%	90%	100%	N/A
Actigraphy	100%	100%	N/A	N/A	N/A	N/A
Exit interview	N/A	N/A	N/A	N/A	N/A	100%

a N/A: not applicable.

Table 2 shows weekly participant completion rate of study assessments. In-lab sleep study and questionnaires were conducted at baseline and postintervention. Sleep logs and heart rate monitoring were conducted during the 4-week intervention. The actigraphy watch was to be worn for baseline week 0 and week 1 of the intervention. An exit interview was conducted postintervention.

### Simple Response Feedback on Acceptability

Regarding the intervention, 70% (7/10) of the participants reported no difficulties to engage in practice, 90% (9/10) of the participants reported that the app was easy to use, and all of them reported that the audios were easy to follow. Regarding the preferred practice time, most of the participants (8/10) preferred shorter practice (less than 20 minutes, with 10‐15 minutes most favored). Most of the participants would like to keep using the app or to practice what they learned at bedtime after they finished the study (Multimedia Appendix 1). The bar graphs present the percentage of participants’ simple answer responses on individual acceptability questions during the qualitative exit interview.

### Open-Ended Qualitative Data

All participants responded to all questions during the exit interviews. We identified multiple themes within 3 major domains. Textboxes 1-3 show representative quotes from participants according to emergent themes. Multiple quotes within each category are from unique individuals.

Textbox 1. **Representative quotes within qualitative domain 1.**

Domain 1: Thoughts or focus of attention during the presleep period as a barrier to sleep

Theme 1.1. Racing, uncontrolled thoughts

“I didn’t have much control over my thoughts. I have an issue where my thoughts would be just coming and passing through my head. I would feel, for example, very sleepy before I go to bed. But then, when I would go to bed, all of a sudden, I would feel awake and then I just kept having racing thoughts, for a very long time to be about work. But then, I would have a lot of random things that would just keep popping in my head.”

“...I would say sometimes I get carried away with thinking about different things. So then it ends up taking longer to fall asleep in general.”

“Just like wandering minds, wandering thoughts...My attention is like wandering very quickly through a lot of different thoughts...With all of those different thoughts, I just can’t get myself to fall asleep.”

Theme 1.2. Loss of control leading to increased stress

“...[I] would think about all the things that happened during the day and would also think about all the things I need to do tomorrow and sometimes I would like to take out my phone and write more things down of like, oh, I’m gonna do this tomorrow. I created to-do things like that.*..*I sometimes try to force myself to not think about anything*...*but I don’t think I was very successful*...*sometimes it would get to a point where if I’m thinking too much about something and still awake then that itself becomes stressful.”

“Focus my attention on my phone.*..*My mind definitely goes through stressful things. I definitely think about work, I think about life situations, and I do definitely get stressed.*..*Sometimes I try to change my mind to make it focus on other stuff. The other times I would just let it run wild and free and let myself worry about those things. I 100% think that where my mind goes kind of dictates how I sleep.”

Theme 1.3. Worry, rumination

“If I’m worrying about something that happens during the day or I’m depressed by something that happens during the day, or I’m worried about something that’s going to happen in the next day, I would find it difficult to fall asleep on that night.”

“*...*once I go to sleep, I start worrying about not sleeping early and how that will impact the following day. And that sort of makes my heart rate of my brain to be more active.”

Textbox 2. **Representative quotes within qualitative domain 2.**

Domain 2: Benefits and skills imparted by mindfulness

Category 2A: Mindfulness specifically at bedtime for sleep

Theme 2A.1. Decreased catastrophizing

“So it made me feel that I shouldn’t be anxious, or wondering, if I’m like, ‘oh, I have to go to sleep or am I going to sleep well or not?’ I just learned that I should let it be. And that’s like I’ll be fine the next day no matter what. Versus how I felt before, like where I thought that like, ‘oh, if I don't sleep well, I’m not going to perform well at work. I'm going to be fatigued. I'm going to feel like this my whole day is going to be ruined.’ So that’s only my perspective about my sleep, sleeping pattern, or just like if I sleep good or not, I just know that I’ll be good the next day.”

Theme 2A.2. Acceptance, nonreactivity, decreased anxiety

“ I don’t have to wait any longer. I can just turn off my lights and turn on the meditation app and it doesn’t matter when I’m going to fall asleep.*..*Instead of like, ‘oh, I need to go and sleep.’ It is just like I’ll go to bed and I’ll meditate and I’ll see where that’s going to get me. If I’m kind of stressed, I would start feeling more relaxed about thinking about my bedtime.”

“Well, I’m trying to tell myself that it’s like, you know, it’s OK if I don’t fall asleep right away. And like if I’m resting, then that’s still good. So I’m like, that’s something I’m trying to do.”

“The app was teaching [me] not worrying about sleep per se. I don’t know how to explain this one, but it was just teaching that you shouldn’t really worry about sleep. You should just be lying on your bed and the sleep would come naturally if you’re not worried about it. And it just gave a lot of practical advice about how to deal with, I don’t know, sleep anxiety is a term. But that feeling of me not having to force myself to sleep and that I can be on bed until I naturally fall to sleep, it seems to be very helpful in like putting me to sleep earlier.”

“I think some of the mindfulness meditation is gonna actually help me find my relationship with sleep in terms of something that you don’t have to continuously struggle or grapple with.*..*Okay, if I fall asleep then okay. If I don’t then that’s okay too. But try to find ways to settle yourself, settle your mind, think about it.”

“ I definitely found that I do fall asleep better, fall asleep easier. And even if I wake up in the middle of the night, I feel less anxious about the next day. So overall, my anxiety about not sleeping well is way, way, way less*...*and I think that quality of my sleep got improved.”

Theme 2A.3. Body awareness and relaxation

“*...*and it definitely helped me out, the meditation helped me out just by relaxing my body a lot. And that’s something, that is already making you feel good when you know that you can really reduce the stress from your body that has been collected maybe in certain parts of your body.”

“The breathing techniques that were taught in the app, I felt they were very helpful and they gave an instant feeling of relaxation*...*And then there was another aspect of trying to focus attention towards specific muscles or specific parts of the body. And I think that’s also very, very helpful.*..*That just somehow made me feel relaxed. I don’t know how, but it made me feel more relaxed and more likely to fall asleep.”

“I think some of the directions given around breath work are quite helpful. Like taking deep breaths, holding them longer and then releasing.”

“*...*it [the practice] definitely made me more aware of sort of where I was holding unnecessary tension. Yeah. And so sometimes it would make me notice that I wasn’t relaxed and then I relax.”

“*...*But focusing on the body, I think it does help because sometimes when they say specifically like relaxing your feet, relaxing your legs, and I would switch my focus to those parts of the body and then try to relax them.”

Theme 2A.4. Self-kindness

“*...*And I think just being told like, it’s some of the common things being common and how like normalizing certain things but then also not like reinforcing certain behaviors but kind of like giving some grace and peace so that you can move beyond some of these normal behaviors that are not great for sleep hygiene.”

“*...*That’s something that if you try to force it more and more then it becomes harder to fall asleep. So you just, kind of, you have a little bit of peace and face to yourself*...*”

Theme 2A.5. Focus on the present

“*...*for example, when I started a new job, I was very, very focused about the next day at work. But that started being less and less throughout the time. So like I was thinking less about work. But I think that the same anxiety pattern played in my head that I think is where my brain kind of had that pattern, like, ‘oh, we go to bed. It’s time to start worrying about something. It’s time to start worrying about job tomorrow.’ Even though my job was not like stressful anymore, my brain would look up for something else to be worried about. So that’s, I think, that is the part that I am now able to change with the mindfulness practice.”

“*...*as time goes on these days, I don’t think about stressful things as much at night before I sleep. I think my coping strategy is, oh, you did enough today. The rest is tomorrow’s problem. And I’ll just battle the issues tomorrow. So like, I would probably briefly think about it. But then I Yeah, but then I would tell myself, okay, we’ll think about tomorrow. Let’s sleep.”

“I think over four or five weeks, [I was] definitely just paying more attention to sleep habits and [my thoughts were like] ‘don’t do this, this can wait till tomorrow.’”

Category 2B: Mindfulness beyond the presleep period impacting sleep

Theme 2B.1. More awareness of sleep habits and sleep hygiene

“...So it definitely has made a difference in terms of sleep habits, too. No more caffeine after 2 p.m. or 2 p.m. to 3 p.m. No more caffeine, like coffee, green tea. So that’s been a big difference there, too. Yeah, I think, in general, I think the mindfulness really helped just relaxing with the music. And so it definitely has improved*...*so I’m always more conscious and mindful about my sleep habits. And so, yeah, it’s made a difference during those five weeks compared to before.”

“It makes me think that sleep is, it is a lifestyle. Sleep, a good quality sleep, not just the quantity of, you know, how many hours, but the quality of sleep, which is going to affect your other lifestyle.”

Theme 2B.2. Improved bedtime routine

“*...*also I mentioned about just managing my time better*...*[I] make sure I brush my teeth, wash my face, do this, do that. So it changes my routine to do that earlier, to not do too many things at night, to also turn off the phone and the laptop, for example, don’t check emails, don’t do this, don’t do that on the computer.*..*It definitely has changed my routine. It is also, a lot less eating, obviously, later in the night. So those are the routines that have changed.”

“So one thing I also wanted to mention is I started to have a more structured and calm approach towards, you know, the end of the day, an hour or two before I sleep.*..*So I go to a different world where I’m reading about something completely different, and then by the time I come to sleep, it seems easier.”

Theme 2B.3. To defuse earlier, not let stress build up to impact sleep at night

“*...*part of the course did focus on how some of the stresses that happened during the day could be carried over during the night, whether we know it or not. So what I began to do is, and this was also suggested in the course, is to keep an eye on your own, on your own psychological health, let’s say during the day. So if you’re getting too worked up on something or too stressed, it’s nice to kind of go away, relax a little bit, so that the stresses don’t accumulate on top of each other and cause an issue during the night. So it’s always good to refresh or empty it out or flush it out, I think, as the course didn’t use the exact word, but just flush it out before you come back, you know, to working on the same thing, be it work or part of your personal life. So I’m kind of keeping an eye on that at the back of my mind.”

Category 2C: Global effects of mindfulness

Theme 2C.1. Increased focus, presence

“I noticed that [the practice] not only affected my focus on my body or breathing during the meditation, but it also enhanced my focus during the day. I noticed that part as well that I am more aware of myself, of the reality of my body during the day. And I can control [my thoughts] easily. If I have a little anxiety going on or anything like that, I’m more aware of being in tune with my body.”

“I want to be consistent with the mindfulness practice because I do see a lot of benefits already from it,*...*my overall awareness did improve a lot. So I can say that my focus not only on work is better, but like focus on everything that I’m doing consciously. It’s like there’s more unconscious stuff that I’m doing. It’s like I can be part of more in tune in my everyday life.”

“*...*I think that maybe even a few minutes of doing that would already change a lot of things throughout my day. So I just like shorter mindfulness practices during the day, I started changing. I think I’ve got more control over many things and then became more aware in everyday situations.”

Theme 2C.2. Calm throughout the day

“The experience itself was quite positive because I think it like brings more like relaxation and like calm to like a certain time of the day and I think it can like last the whole rest of the day or at least last a couple of hours after like doing a little bit of work on this. So even like a couple of minutes, I’ve done it.”

“*...*So I think the mindfulness technique helps, just to take more deep breaths, you know, to really even closing your eyes, like listening to midday music. So it definitely, it definitely has helped me, how to just be more, more focused on just, you know, just like, okay, you know, just to really practicing more mindfulness, calming down, reducing the level of stress during the day.”

Theme 2C.3. Incorporating mindfulness in activities during day

“The Calm app has definitely helped focusing on mindfulness, relaxing more with the mindfulness. I think it also helps even outside of the Calm app, because I have practiced during the day at certain times*...*I think I practice it more, the mindfulness, I even listen to it during the daytime,*...*try to combine the mindfulness music with exercise, such as walking. I think it also helps because at night, it makes me more relaxed, you know, just listening to the Calm app.”

“I think it [mindfulness meditation] allowed me to also slow things down during the day to be more present and to be more mindful about what’s happening in front of me.”

Textbox 3. **Representative quotes within qualitative domain 3.**

Domain 3: Feedback on app use

Category 3A: Preferences and likes

Theme 3A.1. App content

“There are some good things that they talk about, you know, how to reduce stress and anxiety, how to cope with whatever stressful things in life, and how to fall asleep faster, how to calm yourself down. So these are some really good things.”

“I do enjoy it [the app] and I’m definitely going to keep using it. I think that I’ll be using it more.”

Theme 3A.2. Multiple options

“I think what I liked is that there was a breadth of content and there’s multiple different narrators talking about multiple different things. So there’s that variability that can be exercise, maybe to overcome any feeling of, you know, boredom.”

“I think I like the app because there are a lot of options for me to try for the mindfulness practice. And as I was trying different meditations, I also noticed that some meditation might not be very helpful for me and some others are a lot more helpful, I think more useful to me.”

“Overall, I love that I could choose actually, like, every night, a different meditation.”

Theme 3A.3. Diverse voices

“I love the diversity in terms of voices.*..*The app that I had before, I think it was like, only one guy who would take you through meditation. And it could be the same way every time. But this one, [with audios] from different people, you would get different experiences and they would start on a different way and finish on different way or tell you different things. So, you can always learn something new from each one of them. So that’s amazing.”

Theme 3A.4. Varied preferences on the length of soundtracks

“I love the app very much. The only thing, maybe I would add a little bit more options for sleep, maybe shorter options.”

“*...*Like this is just a five minute meditation rather than a full course, which would be, you know, in 10 installments or something*...*I think five to 10 minutes min is preferred for me.”

“So I liked the audios themselves*...*I liked the ‘Seven Days of Sleep’ because I feel like they were a good length for me. I feel like around 15 minutes min, maybe 20, was like my favorite length. But I do also feel like a lot of their meditation are longer, like 30 or 40 minutes min, and I wish there were more variety of the ones that were a little bit on the shorter side. Just so I could have tried those out as well*...*”

“I guess one of the other things I liked is that you could kind of choose how much time you wanted to put into it. And I tried a bunch of different ones.*..*I did like that it was a little shorter. Just because, I mean, the meditation was sort of adding a step into my established bedtime routine. And I didn’t really like doing the super long ones. But it was nice that you could kind of choose.”

“There’s the vast majority of them are like 30 minutes min at least. And I wish I could make it 20 minutes min instead of 30 or 40.”

“I like it to be longer so that I just doze off to sleep while I'm listening to it.”

“*...*Sometimes it takes me longer to fall asleep and I need longer meditation and I never know how long I need and how short I need.”

Category 3B: Challenges and suggestions for improvement

Theme 3B.1. Areas for app improvement

“...I would say that maybe I would sometimes expect more breath work at the beginning of certain meditation...”

“Sometimes...[the practice] would bring me to the point where I can just feel great body sensations and slowly start drifting into sleep. But then I would hear a voice again and I would snap out of sleep.”

“I feel like the user interface was fine, but it felt a little bit cluttered sometimes when you’re like first at the homepage. I feel like they make the sleep tab more relaxed looking, but I feel like when you first open it up, it’s like, okay, there’s all these different things to look at, and it’s a little bit annoying to have to sort of like, okay, first discover and then sleep and then scroll through to find what you’re looking for.”

“Fairly easy to use if you know which program you’re going directly for, I would have liked it to be easier to find individual programs.”

“I think the main thing that I did find to be distracting was when they would take long breaks in the audio where it would, you know, say, okay, now we’re going to leave you to focus on that practice. And sometimes if you were in a sleepy state, you wouldn’t know whether or not it had ended. And then they would start talking again and you would kind of, you know, wake up a little bit. So I found that distracting as opposed to if it had a background music or something to let you know, still going.”

Theme 3B.2. Initial investment for successful app engagement

“I would say it depends on which practice that you choose. Like some of them, perhaps I don’t like that voice that much, or the content that I just did not enjoy that much. And then yeah, at that time when I chose those, I would feel like it’s hard to follow. But after like two or three weeks after I eie,xplore some, [a good] amounts, I found some of those that I think it’s really helpful for me. So then I would just use those repeatedly.”

“I would say*...*before using the Calm app, maybe someone who has experienced using Calm app can kind of talk*...*more about the Calm app,*...*maybe someone who has experienced using it recently and how that has helped him or her or them, I think would be good before using the app. I think overall I’ve been very, very satisfied. One thing I can think about is maybe talk more about the benefits of the Calm app, how to use it. Because a lot of it seems to be, you have to try it on your own. You have to, you know, really learn more on your own. I think it would help to have someone kind of talk about it before starting using it. To have a session, have a mini session maybe, [from] someone with experience using it.”

“*...*probably if I wasn’t obligated to do that [mindfulness meditation], I might have given up in the beginning because it still is an effort. But once you get it into the routine and that barrier is no past, then it’s easy.”

“I think it was difficult to start. I think it got easier over time because it became like a routine, became something that was just part of it, but initially, the first week I think was [difficult].”

Theme 3B.3. Adherence or maintaining engagement

“At the beginning, no [not difficulty to follow], because it was something new. And, you know, my brain was focused on it and I would listen to every word of it and implement it. But over the, you know, the coming weeks, particularly in the last week, because I know exactly what type of routine I’m going to go through. I don’t know, I might be feeling a little bit bored or a little bit, you know, so I’m not so engaged. And I did find my mind started to wander around during the mindfulness practice. So it wasn’t, it wasn’t so easy in the last, in the last week. In the first week, it was much easier.”

“*...*the difficult part is when it’s so easy to follow and I know what the presenter, even though it’s a new presenter or a new narration, I kind of have an idea of where they’re heading towards in terms of the breathing and then focusing on different parts of the body. So it’s nice. It’s easy to follow, but because it’s so easy and I know what’s coming, it might get boring. And if it gets boring, my mind will wander around.”

The first domain is Thoughts or Focus of Attention During the Presleep Period as a Barrier to Sleep. Themes within this domain included the following: 1.1. Racing, uncontrolled thoughts; 1.2. Loss of control leading to increased stress; and 1.3. Worry, rumination (Textbox 1).

The second domain focuses on Benefits and Skills Imparted by Mindfulness. Within this domain, there are 3 categories (Textbox 2). In the first category (2A) Mindfulness Specifically at Bedtime for Sleep, themes included the following: 2A.1. Decreased catastrophizing; 2A.2. Acceptance, nonreactivity, and decreased anxiety; 2A.3. Body awareness and relaxation; 2A.4. Self-kindness; and 2A.5. Focus on the present. Within the second category (2B) Mindfulness Beyond the Presleep Period Impacting Sleep, themes included the following: 2B.1. More awareness of sleep habits and sleep hygiene, 2B.2. Improved bedtime routine, and 2B.3. Defusing stress earlier in the day to avoid impacting sleep*.* Within the third category (2C) Global Effects of Mindfulness, themes included the following: 2C.1. Increased focus, presence, 2C.2. Calm throughout the day, and 2C.3. Incorporating mindfulness in activities during the day.

Within the third domain Feedback on App Use, there were 2 categories (Textbox 3). In the first category (3A) Preferences and Likes, themes included the following: 3A.1. App content, 3A.2. Multiple options, 3A.3. Diverse voices, and 3A.4. Varied preferences on the length of soundtracks. Within the second category (3B) Challenges and Suggestions for Improvement, themes included the following: 3B.1. Areas for app improvement, 3B.2. Initial investment for successful app engagement, and 3B.3. Adherence or maintaining engagement.

### Exploratory Results From Self-Reported Outcomes

**Figure 2.**
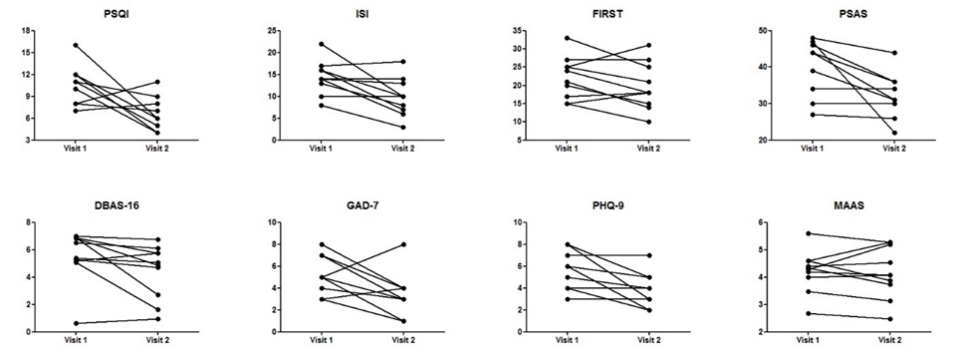
Individual changes on self-reported outcomes at each visit. DBAS-16: Dysfunctional Beliefs and Attitudes about Sleep; FIRST: Ford Insomnia Response to Stress Test; GAD-7: Generalized Anxiety Disorder 7-item; ISI: Insomnia Severity Index; MAAS: Mindful Attention Awareness Scale; PHQ-9: Patient Health Questionnaire–9; PSAS: Pre-Sleep Arousal Scale; PSQI: Pittsburgh Sleep Quality Index.

Based on the self-reported data collected at baseline and postintervention, we observed overall improvement on most of the questionnaire-based outcomes (Multimedia Appendix 1 and Table 3). PSQI scores decreased from 10.6 to 6.9, with a postintervention change of −3.7 (95% CI −6.7 to −0.7), and ISI scores decreased from 14.4 to 9.9, with a postintervention change of −4.5 (95% CI −7.7 to −1.4). Improvement in presleep arousal was indicated by the postintervention change of −7.7 in PSAS scores (95% CI −13.1 to −2.3). Mean scores on anxiety and depression were also decreased, but MAAS scores remained relatively unchanged at the end of the study (Figure 2 and Table 3 ).

**Table 3.** Group-based descriptive statistics of self-reported outcomes at each visit and pre-post changes.

Variables	Baseline (visit 1)		Postintervention (visit 2)		Pre-post changes	
	Mean	SD	Mean	SD	Mean	95% CI
PSQI^a^	10.6	2.6	6.9	2.3	−3.7	−6.7 to −0.7
ISI^b^	14.4	3.8	9.9	4.3	−4.5	−7.7 to −1.4
FIRST^c^	22.2	5.7	19.7	6.4	−2.5	−5.9 to 0.9
PSAS^d^	39.8	7.4	32.1	6.0	−7.7	−13.1 to −2.3
Somatic	13.8	4.2	10.5	2.1	−3.3	−6.0 to −0.6
Cognitive	26.0	4.2	21.6	5.1	−4.4	−7.5 to −1.3
DBAS-16^e^	5.6	1.9	4.4	2.0	−1.1	−2.3 to 0
GAD-7^f^	5.0	1.8	3.5	2.0	−1.5	−3.3 to 0.3
PHQ-9^g^	5.9	1.9	3.8	1.5	−2.1	−3.6 to −0.7
MAAS^h^	4.21	0.77	4.16	0.94	−0.05	−0.43 to 0.32

a PSQI: Pittsburgh Sleep Quality Index.

b ISI: Insomnia Severity Index.

c FIRST: Ford Insomnia Response to Stress Test.

d PSAS: Pre-Sleep Arousal Scale.

e DBAS-16: Dysfunctional Beliefs and Attitudes about Sleep.

f GAD-7: Generalized Anxiety Disorder 7-item.

g PHQ-9: Patient Health Questionnaire–9.

h MAAS: Mindful Attention Awareness Scale.

## Discussion

### Principal Findings

As the first step toward understanding the effectiveness and mechanisms of bedtime MM practice for insomnia, this study was focused on feasibility and acceptability of app-guided bedtime MM practice with in-lab and at-home sleep assessments. We recruited 13 participants and retained 10 (77%) to complete the study protocols within 8 months. We achieved our target rates (90%‐100%) with respect to completion of questionnaires, daily sleep log, heart rate and actigraphy data collection, daily practice adherence, and exit interview supporting the feasibility of a larger study with these procedures [47].

Overall, most participants reported that the app was easy to use and app-guided MM was helpful. Bedtime practice not only helped with sleep but skills learned also translated to and were helpful at other times during the day. Multiple cognitive-emotional processes were mentioned, including decreased catastrophizing, increased acceptance and nonreactivity, overall body awareness, decreased anxiety and letting go of worry, normalizing, and focusing on the present. We also gained valuable feedback from participants regarding future adaptations that might be considered. Future iterations may consider preferred lengths of bedtime MM practice, instructions around use of ear pods, more user-friendly app interface, and customizations based on other comorbidities such as chronic pain.

We observed clinically meaningful improvements in sleep quality (indicated by PSQI score), insomnia severity (indicated by ISI score), presleep arousal (indicated by PSAS score), vulnerability to sleep disruption (indicated by FIRST), and dysfunctional beliefs about sleep (indicated by DBAS score). Interestingly, we only found very small changes in MAAS score after the intervention. Several possible explanations include heterogeneity of the study subjects and small sample size, short duration of the intervention, and a relevance mismatch between questions on MAAS and our intervention. Other studies have also reported an insensitivity of MAAS in response to mindfulness exposures (lots of references on this). Future studies might consider other measures of mindfulness, for example, the Multidimensional Assessment of Interoceptive Awareness [48,49].

Telehealth and technology-based approaches can improve accessibility to health care. Given the increasing number of studies with remotely delivered mindfulness-based interventions [50-57], we designed this study to include remote intervention and home assessments. Although data collection through wearables can decrease participants’ burden associated with scheduling, travel, costs, and wait time, structured demonstration and detailed instructions are important to ensure successful data collection. In our study, we found that active investigator demonstration with the participant prior to leaving the visit was critical to success, even in a relatively young, technology-literate population. The acceptability and feasibility of such app-based intervention and assessment with wearable technology in other populations, for example, older populations, or otherwise less tech-savvy or lower literacy, remain unclear.

We identified gaps and needs to be addressed for future larger-scale studies. For example, while we acknowledge that using electronic devices in bed is contradictory to current standard sleep hygiene recommendations, a large number of patients already follow standard recommendations but still greatly experience difficulty falling asleep. While our study developed targeted instructions for the app-based intervention (eg, use only audios, no or minimal screen exposure, dim light, do-not-disturb mode, and auto-stop playing), optimal app use regarding the timing and duration of practice, as well as the choice of audios to meet individual preferences, should be further studied.

Exploratory analysis of pre- to postsubjective measures supports the potential role for MM in the improvement of sleep, insomnia severity, and presleep arousal [22,58-62]. Recent RCTs in varied populations support community dissemination of the Calm app for sleep disturbance [47]. [63], Several RCTs have shown that the use of the Calm app decreased fatigue, daytime sleepiness, and cognitive and somatic arousal and improved depression and anxiety symptoms [47,63,64]. Our line of inquiry is the first to probe a deeper understanding of efficacy and mechanisms of app-based MM practice specifically at bedtime.

### Limitations and Future Research

One limitation of this study is that population composition may not reflect sociodemographic profiles of the general population. Our final participant sample was relatively younger (aged 22‐50 years), healthy, well-educated, and with chronic insomnia, albeit less severe upon baseline assessment. While chronic insomnia disorder is commonly diagnosed in women, we excluded breastfeeding women or those currently with a young baby, due to presumed irregular circadian rhythms common in this population. Previous research also demonstrates differences between children or adolescents and adults with respect to circadian rhythmicity, causes of insomnia, wake-sleep schedules, and scoring rules for sleep studies; thus, we excluded children. Similarly, as we age, the structure of sleep and the functionality of brain regions change drastically, and physiological changes associated with mindfulness practice in older adults may be different from younger adults. Thus, older adults were also not included in this protocol. In addition, this was a proof-of-concept uncontrolled, single-arm study in a relatively younger and healthier population. The feasibility of retaining a control arm, implementing such a study in other populations (eg, older adults or with comorbid conditions), remains unclear.

 In this feasibility pilot study, we prioritized evaluating engagement and adherence rather than enforcing strict adherence to a bedtime-only meditation schedule. While we instructed participants to practice at bedtime, we allowed them to explore the app at other times of the day to understand the naturalistic use of the app, which is relevant for real-world implementation. Although the majority of participants practiced only during bedtime, we acknowledge that the flexibility introduces variability in exposure, making it challenging to attribute outcome changes solely to bedtime mindfulness practice.

It is also important to emphasize that we were explicitly aware of the impact of digital devices and light exposure at bedtime and therefore carefully developed instructions and tips to minimize risks but acknowledge that further refinement of instructions is needed. This is especially critical for potential future RCTs with a larger sample size.

To our knowledge, this is the first study to primarily target bedtime mindfulness practice using remote intervention and to collect data from comprehensive assessments including self-reported outcomes, in-lab sleep study, at-home assessment, and qualitative data from interviews. Despite the limitations, this feasibility study informs and inspires future investigation to better understand neurophysiological mechanisms of bedtime mindfulness practice. In addition, future studies are encouraged to refine instructions for optimal intervention, to collect comprehensive subjective and objective data, and to include a diverse group of patients with insomnia with broader eligibility criteria.

### Conclusions

The feasibility outcomes met our preset criteria for success; therefore, a study with bedtime app-guided MM as an intervention in patients with insomnia and the hybrid design with in-lab and at-home assessments is feasible. Our exploratory analysis of subjective measures indicated improvement in sleep, insomnia severity, and presleep arousal.

## Supplementary material

10.2196/67366 Multimedia Appendix 1 Participants’ feedback on bedtime app–based mindfulness intervention acceptability.

## Abbreviations

CONSORT: Consolidated Standards of Reporting Trials
DBAS-16: Dysfunctional Beliefs and Attitudes about Sleep
*DSM-IV*: *Diagnostic and Statistical Manual of Mental Disorders* (Fourth Edition)
*DSM-V*: *Diagnostic and Statistical Manual of Mental Disorders* (Fifth Edition)
FIRST: Ford Insomnia Response to Stress Test
ISI: Insomnia Severity Index
MAAS: Mindful Attention Awareness Scale
MM: mindfulness meditation
PSAS: Pre-Sleep Arousal Scale
PSG: polysomnograph
PSQI: Pittsburgh Sleep Quality Index
RCT: randomized controlled trial
REDCap: Research Electronic Data Capture
