# Supplementary figures and images for: Bedtime App–Guided Mindfulness Meditation in Patients With Insomnia: Mixed Methods Feasibility and Acceptability Pilot Study
Source: JMIR Form Res. 2025 Sep 30;9:e67366. doi: 10.2196/67366 (PMC12483474; doi:10.2196/67366)

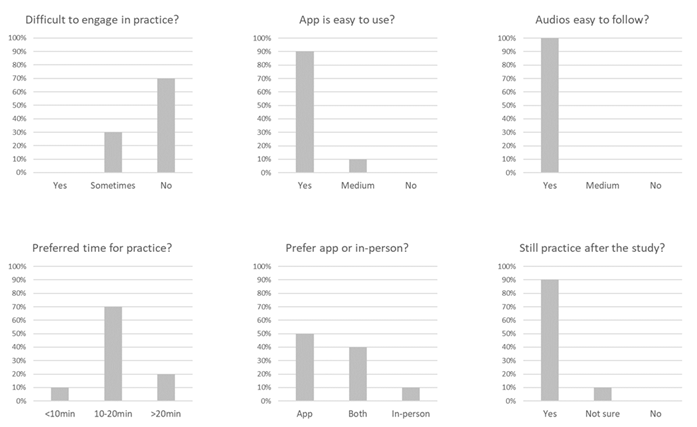

Supplement: Multimedia Appendix 1 [file formative-v9-e67366-s001.png]
